# Dogs with advanced myxomatous mitral valve disease have evidence of gastrointestinal bacterial translocation and systemic inflammation

**DOI:** 10.1371/journal.pone.0337580

**Published:** 2025-11-24

**Authors:** Maria C. Jugan, Gabrielle A. Rands, Joerg M. Steiner, Natalia Cernicchiaro, Matthew C. Tanner, Lillian M. Novotny

**Affiliations:** 1 Department of Clinical Sciences, College of Veterinary Medicine, Kansas State University, Manhattan, Kansas, United States of America; 2 Gastrointestinal Laboratory, Department of Small Animal Clinical Sciences, College of Veterinary Medicine and Biomedical Sciences, Texas A&M University, College Station, Texas, United States of America; 3 Center for Outcomes Research and Epidemiology and Department of Diagnostic Medicine/Pathobiology, College of Veterinary Medicine, Kansas State University, Manhattan, Kansas, United States of America; Medizinische Universitat Graz, AUSTRIA

## Abstract

This study aimed to explore the potential relationship between the severity of cardiovascular disease and evidence of gastrointestinal bacterial translocation with systemic and cardiac inflammation in dogs with myxomatous mitral valve disease (MMVD). Thirty-six client-owned dogs, including 25 dogs with American College of Veterinary Internal Medicine Stage B1 or B2 disease but without increased left atrial pressure (Group 1) and 11 dogs with stage B2 disease with increased left atrial pressure or stage C disease (Group 2), were enrolled prospectively with an echocardiographic diagnosis of untreated MMVD. Serum lipopolysaccharide (LPS), interleukins (i.e., IL-2, IL-6, IL-8), tumor necrosis factor-alpha, and cardiac troponin were measured at enrollment. Presence of gastrointestinal clinical signs, fecal scores, body condition score, and muscle condition score were recorded. Linear regression models were used to compare LPS and inflammatory biomarkers based on MMVD severity. Spearman’s correlation was used to evaluate possible associations between inflammatory markers and LPS. The Fisher Exact test was used to compare proportions of dogs with gastrointestinal signs between Groups 1 and 2. Twenty percent of dogs in Group 1 had gastrointestinal clinical signs compared to 66.7% in Group 2 (P < 0.0001). Serum IL-6 (P = 0.037) and LPS (P = 0.024) concentrations were significantly associated with MMVD stage severity. Serum LPS and IL-6 were positively correlated (r_s_ = 0.81; P < 0.0001). This study indicates that dogs with MMVD have evidence of loss of gastrointestinal barrier function as evidenced by bacterial translocation as the disease progresses in severity, which may be associated with systemic inflammation. These findings warrant further evaluation of gastrointestinal barrier function and maybe even the gastrointestinal microbiome as therapeutic targets in dogs with MMVD.

## Introduction

Myxomatous mitral valve disease (MMVD) is diagnosed in 75% of dogs with acquired cardiovascular disease (CVD) [1,2], with a 50 - > 90% prevalence reported in some breeds [1–4]. Once dogs with MMVD experience heart failure (CHF), reported survival times range from approximately 6–33 months [5–7]. Gastrointestinal (GI) hyperpermeability is a well-recognized co-morbidity in humans with CVD. The gut-heart axis is interconnected, with disturbances in one system leading to progressive dysfunction in the other [8]. Although there are no published studies specifically designed to assess GI signs in dogs with CVD, diarrhea has been reported in up to 14% dogs with CHF [9]. One study in dogs demonstrated GI dysbiosis patterns similar to those in humans with CHF [10,11].

The interrelation between GI barrier function and CVD is complex. Chronic CVD results in decreased GI mucosal perfusion due to physiologic shunting of blood away from the splanchnic circulation, leading to GI ischemia [12,13]. Additionally, increased GI thickness, secondary to inflammation and edema, is noted in humans with CHF [14]. Consistent with these findings, increased blood biomarkers of GI barrier dysfunction [e.g., lipopolysaccharide (LPS), LPS-binding protein, trimethylamine N-oxide] have been documented in human CVD patients, with greater elevations with more severe disease [15–19]. Concurrent with loss of barrier function, increased GI permeability and GI malabsorption have also been demonstrated by increased sugar permeability and decreased sugar absorption [14,19,20].

In a self-perpetuating cycle, circulating enteric metabolites and bacterial pathogen-associated molecular patterns lead to systemic inflammation. Lipopolysaccharide, in particular, stimulates pro-inflammatory cytokine release [e.g., tumor necrosis factor (TNF)-α, interleukin (IL)-1β, IL-6] through toll-like receptor binding. While serum LPS concentrations have not been evaluated in the context of canine CVD, elevations have been noted in diseases associated with GI hyperpermeability in dogs [21,22]. Inflammation driven by LPS and other pro-inflammatory cytokines increases the risk of CVD development and progression in humans [17–20,23–26]. Although there have been fewer evaluations of long-term impact of loss of GI barrier function on the progression of CVD in dogs, evidence of systemic inflammation has been documented in some dogs [27–29]. Endotoxemia also results in decreased GI microbiota that help maintain the GI barrier through direct enteroendocrine signaling [30]. Therefore, endotoxemia alone could exacerbate loss of GI barrier function. In addition to GI malabsorption documented in humans with CVD, higher circulating LPS concentrations are noted in humans with cardiac cachexia [15]. Importantly, cachexia is related to increased mortality in both humans and dogs with CVD [31–33].

Documenting a clinically relevant association between CVD severity and loss of GI barrier function would advance the understanding of the gut-heart axis in dogs. It would also underscore the need to evaluate impact of GI barrier function on disease outcome and effectiveness of GI-targeted therapies in CVD management. Therefore, the objective of the study was to evaluate the association between stages of CVD, GI barrier function, as estimated by evidence of GI bacterial translocation via endotoxemia, and systemic inflammation via serum cytokines in client-owned dogs with untreated MMVD. We hypothesized that serum LPS concentrations would be higher in dogs with more severe MMVD, and secondarily, that LPS would correlate positively with biomarkers of systemic inflammation.

## Materials and methods

Adult client-owned dogs were enrolled prospectively and consecutively at the time of initial evaluation and diagnosis of MMVD at the Kansas State University Veterinary Health Center (KSU VHC). The sample size required to detect the effect of interest, assuming mean serum LPS concentrations of 0.74 + /- 0.45 EU/mL in human patients with CHF versus 0.37 + /- 0.23 EU/mL with stable CVD [16], α = 0.05, and β = 0.8, was a total study population of 50 MMVD dogs, including 25 dogs with stage B1 or B2 disease without increased left atrial pressure (Group 1) and 25 dogs with either Stage B2 disease and increased left atrial pressure or stage C disease (Group 2).

Mitral valve disease and stage classification was diagnosed based on ACVIM consensus guidelines following echocardiogram interpretation by a board-certified veterinary cardiologist [34]. In dogs with CHF, diagnosis was supported by thoracic radiographs. Dogs were required to have baseline blood work, including a complete blood count and chemistry profile, within 3 months of enrollment. Dogs with concurrent systemic disease or receiving medications or supplements aside from routine preventatives within the month prior to enrollment were excluded; a single dose of trazadone or gabapentin prior to the appointment was allowed. In dogs with CHF, a single dose of furosemide and/or pimobendan were permitted prior to enrollment.

A subset of healthy, client-owned adult dogs were enrolled from pets belonging to staff and students of the VHC to determine baseline outcome concentrations (i.e., LPS, cytokines). Dogs were determined to be healthy based on physical examination, screening questionnaire for absence of systemic or GI signs, and normal baseline blood work. Healthy control dogs had not received any medications aside from routine preventatives within the previous six months. A prescription diet to control historical GI signs or raw diets or treats were conditions for exclusion in all dogs (i.e., MMVD and healthy controls).

At the time of presentation, blood was drawn for measurement of serum LPS (canine-specific ELISA; MyBiosource, MyBiosource, Inc, San Diego, California, USA) [21,35,36], IL-2, IL-6, IL-8, and TNFα (MSD multiplex system; Meso Scale Discovery Multi-Spot Assay System; Canine Proinflammatory Panel 3 Assay Ultra-Sensitive Kit; Texas A&M GI Laboratory) [37–39], and canine troponin (Advia Centaur CP High-Sensitivity Troponin; Texas A&M GI Laboratory) [40]. Blood was allowed to clot at room temperature, centrifuged, serum separated, and frozen (−80⁰C) immediately after serum separation, and all assays performed at study completion. Lipopolysaccharide ELISA was performed in-house according to manufacturer instructions, with all samples analyzed in duplicate fashion. Samples with gross hemolysis or lipemia following recentrifugation were excluded from analysis. Fecal scoring (FS) based on a 1–5 scoring system where 1 = dry, hard stool and 5 = liquid, with no solid fecal material was performed on fresh, naturally defecated fecal samples at the time of enrollment by a single investigator (MCJ) or by showing owners a picture-based chart of the scoring system when feces were not able to be obtained [41]. For the purposes of this study, a FS ≥ 3.5 was considered diarrhea. A single investigator (MCJ) performed body condition scoring (BCS) on a 1–9 scale and muscle condition scoring (MCS; normal, mild, moderate, severe) based on previously developed criteria [42,43]. Assessments for FS, BCS, and MCS were performed prior to completion of the echocardiogram report. Owners also completed a questionnaire based on a previously developed scoring system for dogs with chronic inflammatory enteropathies to document presence and severity of GI clinical signs [44]. (**Questionnaire in** S1 Questionnaire). All procedures were approved by the KSU Institutional Animal Care and Use Committee (protocol IACUC-4641).

### Statistical analysis

Statistical analyses were performed using commercially available software (GraphPad Prism Version 10.1.2, GraphPad Software, Boston, Massachusetts, USA and Stata 17.0, StataCorp, College Station, Texas, USA). Demographic characteristics of the study population, serum LPS, cytokines, and troponin concentrations were assessed for normality using the Shapiro-Wilk test. As most parameters were not normally distributed, most parameters, as well as reproductive status (e.g., neutered versus intact), age, and BCS, were compared between Groups 1 and 2, as well as MMVD versus healthy control dogs, using the Mann-Whitney U test. For results below the lower limit of detection of the assay, the values of the minimally detectable value were reported: 20 pg/mL (IL-2), 20 pg/mL (IL-6, IL-8), 4.8 pg/mL (TNF-α), and 20 pg/mL (troponin) and used for statistical analysis. Spearman (r_s_) correlation coefficient between markers of GI translocation, inflammation, and heart enlargement as defined by the ratio of the left atrial to aortic root diameter [LA:Ao] was calculated. Strength of correlation was defined as previously (0–0.09 = negligible, 0.1–0.39 = weak, 0.4–0.69 = moderate, 0.7–0.89 = strong, 0.9–1.0 = very strong) [45]. Bonferroni correction was used to adjust the *P* value level of significance for multiple comparisons (level noted with respective results). The Fischer Exact test was used to compare the proportion of dogs with GI clinical signs between MMVD Groups 1 and 2.

The associations between MMVD severity stage groups, along with other exposures of interest, with serum concentrations of LPS, inflammatory cytokines, and cardiac troponin were evaluated using linear regression models. The outcomes included serum concentrations of LPS, cytokines (IL-2, IL-6, IL-8, and TNF-α), and cardiac troponin, all originally recorded on a continuous scale. Exposures of interest included MMVD severity stage, breed, BCS, age, weight, and MCS. Breeds were categorized into three groups consisting of toy or small, medium, and large/giant breeds [46,47]. Given the sparsity of data in one category, BCS was grouped into categories (BCS_cat): 0 = 2, 3, 4; 1 = 4; 2 = 6; and 3 = 7. Linear regression models with a Gaussian distribution and an identity link were built. Initially, univariable associations between MMVD severity stage, the main exposure of interest, age, weight, BCS, sex, MCS, and breed category with each outcome were assessed. The linearity assumption for continuous exposures (age and weight) was tested by fitting a locally weighted regression of the fitted values of the outcome against each exposure. Confounding was evaluated by sequentially incorporating a priori confounders (age, weight, and breed) and assessing whether their inclusion altered the magnitude of the association between MMVD severity stage and each outcome by more than 30%. Log_10_ transformations were attempted to improve model assumptions for serum concentrations of IL-2, IL-8, troponin, and TNF-α. Evaluation of residual diagnostics, including tests for homoscedasticity and normality, were conducted using both graphical and statistical (Breusch-Pagan/Cook-Weisberg and Shapiro-Wilk, respectively) methods. Back transformation of log_10_-transformed values was performed to ensure consistency in reporting. Due to the small sample size (n = 28–36), multivariable models beyond bivariable analysis could not be fitted. Marginal means, along with their 95% confidence intervals and overall *P* values, are presented. Statistical significance was set at P* *< 0.05.

## Results

### Study populations

Thirty-six dogs with MMVD were enrolled prospectively between August 2022 and November 2023, including 25 dogs in group 1 and 11 dogs in group 2. MMVD dogs included 19 castrated males (n = 13 Group 1; n = 6 Group 2) and 17 spayed females (n = 12 Group 1; n = 5 Group 2). Breeds included in the MMVD population were chihuahua (n = 8), mixed breeds (n = 5), dachshund (n = 3), Yorkshire terrier (n = 3), Australian heeler (n = 2), Cavalier King Charles spaniel (n = 2), schnauzer (n = 2), and 1 each Australian shepherd, bichon frise, Labrador retriever, miniature poodle, papillon, Pekingese, Pomeranian, rat terrier, shih tzu, toy Australian shepherd, and toy fox terrier. Median age was 11.0 years (range, 4.9–17.3 years), median weight was 5.2 kg (range, 1.9–36.2 kg), median BCS was 5.5 out of 9 (range, 2–7), and median MCS was normal (0; range, normal to moderate muscle atrophy [0–2]). There was no difference in age (P = 0.230), weight (P = 0.741), BCS (P = 0.516), or MCS (P = 0.253) between group 1 and group 2 MMVD dogs.

The healthy control group included 10 dogs, enrolled prospectively between November 2022 and January 2023, and was comprised of 1 intact male, 7 castrated males, 1 intact female, and 1 spayed female. Breeds included dachshund (n = 3), presa canario (n = 2), mixed breeds (n = 2), and 1 each German shepherd, French bulldog, and pitbull terrier. Median age was 5.4 years (range, 1–8 years), median weight was 27.6 kg (range, 5.1–55.4 kg), median BCS was 5.5 (range, 4–7), and MCS was normal for all dogs. Healthy control dogs were significantly younger (P < 0.001) and weighed more (P < 0.01) than MMVD dogs, but there was no difference in BCS (P = 0.889) or MCS (P = 0.187) between groups.

### Gastrointestinal clinical signs

Per inclusion criteria, none of the healthy control dogs had a history of GI clinical signs. Within the MMVD group, 8 dogs (all within Group 2) had a reported decrease in appetite, ranging from a mild to a severe decrease. Diarrhea was reported in 3 dogs (n = 1 Group 1; n = 2 Group 2), ranging from slightly soft to very soft consistency (FS 3.5–4.0). The dog in Group 1 that exhibited diarrhea had slightly soft fecal consistency, and an increase in stool frequency (2–3 times per day) was also reported. Weight loss was documented in 8 dogs (n = 3 Group 1; n = 5 Group 2), ranging from 5 to >10% body weight. One MMVD dog (Group 2) reported a history of vomiting once per week. Overall, dogs in Group 1 (20%) were less likely to have GI clinical signs compared to those in Group 2 (66.7%; Relative risk 0.22, 95% CI 0.096–0.460; P < 0.0001).

### Biomarkers of bacterial translocation and inflammation

#### Biomarkers in MMVD versus healthy control dogs.

Median serum troponin concentrations were significantly higher in MMVD dogs (96 pg/mL; range, 20–1610 pg/mL) than in healthy control dogs (25 pg/mL; range, 20–76 pg/mL; P < 0.001; Bonferroni corrected *P* value for significance* *< 0.003). There were no other significant differences in serum LPS or cytokine concentrations between the entire MMVD study population and healthy control dogs. This remained true when values were compared based on MMVD group (i.e., Group 1 or 2 separately versus healthy control dogs; **Table in** **S2 Table****).**

#### Biomarkers and MMVD severity.

Table 1 depicts the results of the descriptive statistics for serum concentrations of all evaluated cytokines, serum LPS and troponin by MMVD disease severity stage. Tables 2 and 3 present the mean estimates for associations between the main exposure of interest (MMVD disease severity stage), and other exposures listed above, with the different study outcomes based on univariable models.

**Table 1:** Descriptive Statistics of Serum Concentrations of Cytokines, Lipopolysaccharides and Cardiac Troponin by Severity Stage in 36 Client-owned Dogs with Untreated Myxomatous Mitral Valve Disease (MMVD).

	Statistic	Outcomes (units)					
		IL-2 (pg/mL)	IL-6 (pg/mL)	IL-8 (pg/mL)	TNF-α (pg/mL)	LPS (ng/mL)	Troponin (pg/mL)
**MMVD disease severity stage** ^**a**^	Mean	83	65	3924	10.3	6.2	77
1 (B1/B2 normal pressure)	Median	69	55	3484	7.9	5.5	52
	SD	54	42	2640	6.4	2.6	54
	Min	20	12	198	4.8	1.3	20
	Max	215	176	11504	25.3	12.4	199
	N	25	25	25	25	20	25
2 (B2 high pressure/C)	Mean	120	110	3816	12.1	8.8	602
	Median	50	106	1603	4.8	93.3	371
	SD	125	82	4498	12.4	2.4	465
	Min	20	14	144	4.8	4.3	111
	Max	351	251	12242	41.2	11.3	1610
	N	11	11	11	11	8	11
*P-*value		0.973	0.220	0.276	0.424	0.047	<0.001
Total	Mean	94	79	3891	10.9	7.0	237
	Median	69	61	3231	6.7	6.4	96
	SD	82	60	3250	8.5	2.7	352
	Min	20	12	144	4.8	1.3	20
	Max	351	251	12242	41.2	12.4	1610
	N	36	36	36	36	28	36

^a^MMVD disease severity stage group 1: Stage B1: Mitral or tricuspid valve regurgitation but no echocardiographic remodeling [left atrial (LA) or ventricular (LV) enlargement]; Stage B2 (early): Mild to moderate LA enlargement but without increased LA pressure, normal to impaired LV filling, no congestive heart failure (CHF). Group 2: Stage B2: Moderate to severe LA enlargement with increased LA pressure, normal to impaired LV filling, no CHF; Stage C: Current or past signs of CHF caused by MMVD.

SD = Standard deviation; N = number of observations.

*P-*value notes comparison between MMVD severity stages. Bonferroni corrected *P-*value for significance <0.008.

**Table 2:** Association between Exposures of Interest with Serum Cytokine Concentrations Based on Univariable Models‡ in 36 Client-owned Dogs with Untreated Myxomatous Mitral Valve Disease (MMVD).

		Cytokine Outcomes, units			
Exposure, units	N	IL-2^†^ (pg/mL)	IL-6 (pg/mL)	IL-8^†^ (pg/mL)	TNF-α^†^ (pg/mL)
MMVD disease severity^a^		P = 0.944	P = **0.037**	P = 0.241	P = 0.865
1 (B1/B2 normal pressure)	25	66 (46-93)	65 (42-88)	,951 (950-4571)	8.7 (7.8-11.5)
2 (B2 high pressure/C)	11	68 (40-114)	110 (75-145)	1862 (1000-3548)	8.5 (5.6-12.6)
Age, years	36	P = 0.155 1.1 (0.8, 1.2)	P = 0.099 5.1 (−1.0, 11.1)	P = 0.950 1.0 (0.9-1.1)	P = 0.373 1.0 (1.0-1.1)
Weight, kg	36	P = **0.007** 1.0 (0.9, 1.0)	P = 0.090 −1.8 (−3.8, 0.30)	P = 0.147 1.0 (0.9-1.0)	P = **0.031** 1.0 (1.0-1.0)
Breed categories		P = **0.036**	P = 0.209	P = 0.410	P = 0.182
1 (Toy or small)	30	78 (58-105)	84 (64-108)	2884 (1950-4266)	9.6 (7.4-12.0)
2 (Medium)	1	20 (4-102)	17 (−103, 137)	1148 (135-10000)	4.8 (1.4-17.4)
3 (Large or giant)	5	32 (16-68)	44 (−9, 98)	1622 (617-4266)	5.8 (3.2-10.2)
Sex		P = 0.825	P = 0.839	P = 0.551	P = 0.830
1 (male castrated)	19	68 (46-102)	77 (48-105)	2344 (1413-3802)	8.5 (6.3-11.5)
3 (female spayed)	17	65 (42-98)	81 (51-111)	2884 (1698-4898)	8.9 (6.5-12.3)
Body condition score (1–9 scale)		P = 0.601	P = 0.950	P = 0.507	P = 0.618
2–4	11	76 (45-129)	84 (46-123)	2188 (1148-4266)	9.3 (6.3-14.1)
5	7	45 (23-87)	68 (20-116)	1698 (759-3890)	6.8 (4.0-11.0)
6	9	66 (36-120)	82 (40-125)	3548 (1738-7244)	10.0 (6.5-15.9)
7	9	75 (42-138)	77 (34-119)	3090 (1514-6310)	8.3 (5.3-12.9)
Muscle condition score		P = 0.357	P = 0.736	P = 0.160	P = 0.410
0 (normal)	22	71 (49-102)	79 (53-106)	3020 (1950-4786)	8.9 (6.8-12.0)
1 (mild)	11	50 (30-85)	71 (33-109)	1585 (851-2951)	7.2 (4.9-10.7)
2 (moderate)	3	107 (39-288)	102 (30-174)	4467 (1349-14791)	12.6 (5.9-26.9)

^‡^From univariable linear regression models using a Gaussian distribution with an identity link, each outcome was tested against a single fixed effect at a time.

Data are expressed as mean (95% confidence interval); P-values represent the overall significance based on the F-test. P-values in bold depict significant associations (P < 0.05).

^a^MMVD disease severity stage group 1: Stage B1: Mitral or tricuspid valve regurgitation but no echocardiographic remodeling [left atrial (LA) or ventricular (LV) enlargement]; Stage B2 (early): Mild to moderate LA enlargement but without increased LA pressure, normal to impaired LV filling, no congestive heart failure (CHF). Group 2: Stage B2: Moderate to severe LA enlargement with increased LA pressure, normal to impaired LV filling, no CHF; Stage C: Current or past signs of CHF caused by MMVD.

N = number of observations. † Back-transformed values from a log_10_ distribution.

**Table 3:** Association between Exposures of Interest with Serum Lipopolysaccharide and Troponin Concentrations Based on Univariable Models‡ in 36 Client-owned Dogs with Untreated Myxomatous Mitral Valve Disease (MMVD).

		Outcomes	
Exposure, units	N	LPS (ng/mL)	Troponin^†^ (pg/mL)
MMVD disease severity^a^		P = **0.024**	P **< 0.001**
1 (B1/B2 normal pressure)	25	6.2 (5.1-7.4)	62 (48-83)
2 (B2 high pressure/C)	11	8.8 (6.9-10.6)	447 (282-692)
Age, years	36	P = 0.166 0.2 (−0.1, 0.5)	P = **0.047** 1.05 (1.00-1.26)
Weight, kg	36	P = 0.921 0.01 (−0.1, 0.1)	P = 0.229 1.0 (1.0-1.1)
Breed categories		P = 0.788	P = 0.267
1 (Toy or small)	30	7.0 (5.8-8.2)	102 (66-158)
2 (Medium)	1	5.2 (−0.7, 11)	49 (5-525)
3 (Large or giant)	5	7.4 (4.4-10.3)	234 (81-676)
			
Sex		P = 0.689	P = 0.382
1 (male castrated)	19	7.2 (5.6-8.7)	132 (76-229)
3 (female spayed)	17	6.7 (5.2-8.3)	93 (52-166)
Body condition score (1–9 scale)		P = 0.230	P = 0.682
2–4	11	8.5 (6.7-10.3)	135 (65-282)
5	7	6.0 (3.7-8.2)	145 (58-363)
6	9	6.6 (4.3-8.8)	78 (34-174)
7	9	6.2 (4.1-8.2)	107 (48-240)
Muscle condition score		P = 0.468	P = 0.430
0 (normal)	22	6.5 (5.1-7.9)	96 (56-158)
1 (mild)	11	7.9 (6.0-9.8)	166 (81-339)
2 (moderate)	3	6.8 (2.8-10.8)	87 (22-347)

^‡^From univariable linear regression models using a Gaussian distribution with an identity link, each outcome was tested against a single fixed effect at a time.

Data are expressed as mean (95% confidence interval); P-values represent the overall significance based on the F-test. P-values in bold depict significant associations (P < 0.05).

^a^MMVD disease severity stage group 1: Stage B1: Mitral or tricuspid valve regurgitation but no echocardiographic remodeling [left atrial (LA) or ventricular (LV) enlargement]; Stage B2 (early): Mild to moderate LA enlargement but without increased LA pressure, normal to impaired LV filling, no congestive heart failure (CHF). Group 2: Stage B2: Moderate to severe LA enlargement with increased LA pressure, normal to impaired LV filling, no CHF; Stage C: Current or past signs of CHF caused by MMVD.

N = number of observations.

^†^Back-transformed values from a log_10_ distribution.

Based on univariable analyses, the stage of MMVD severity was significantly associated with LPS, IL-6, and troponin outcomes. Serum IL-6 concentrations were significantly higher in dogs in the MMVD B2 high pressure/C disease group (Group 2; 110 pg/mL) compared to those in the B1/B2 normal pressure group (Group 1; 65 pg/mL; P = 0.037). Similarly, dogs in MMVD Group 2 had significantly higher serum LPS (8.8 ng/mL) and troponin (447 pg/mL) concentrations than those in Group 1 (6.2 ng/mL and 62 pg/mL, respectively) (P = 0.024 and <0.001, respectively). Serum IL-2, IL-8, and TNF-α concentrations did not significantly differ by MMVD disease severity stage. Age, weight, and breed were not identified as confounders of the association between MMVD severity stage and these outcomes, nor did they remain significant exposures in bivariable models.

Table 4 depicts the correlation analyses between serum biomarkers of GI translocation, systemic inflammation, and LA:Ao. Serum LPS and IL-6 concentrations were strongly, positively correlated (r_s_ 0.81, 95% CI 0.64–0.91; P < 0.0001; Bonferroni corrected *P* value for significance < 0.006).

**Table 4:** Spearman correlation coefficient (r_s_) between echocardiographic measurements (i.e., left atrial:aortic root diameter; LA:Ao), markers of loss of GI barrier function (i.e., lipopolysaccharide; LPS), and cardiac (i.e., troponin) and systemic (i.e., cytokines) inflammation in 36 dogs with untreated myxomatous mitral valve disease (MMVD).

Variables	r_s_	95% CI	P value
La:Ao and IL-6	0.08	−0.30 to 0.45	0.66
La:Ao and LPS	0.11	−0.34 to 0.52	0.62
La:Ao and TNF-α	−0.18	−0.52 to 0.21	0.34
Troponin and IL-6	0.16	−0.19 to 0.47	0.36
Troponin and LPS	0.20	−0.19 to 0.54	0.30
Troponin and TNF-α	−0.09	−0.41 to 0.26	0.61
LPS and IL-2	−0.27	−0.59 to 0.13	0.17
LPS and IL-6	0.81	0.64 to 0.91	**<0.0001**
LPS and TNF-α	−0.45	−0.71 to −0.09	0.01

CI, confidence interval; IL, interleukin; TNF, tumor necrosis factor. A Bonferroni corrected *P* value of <0.006 was considered significant (bold).

## Discussion

In this study, we evaluated GI clinical signs in conjunction with systemic inflammatory biomarkers and LPS, as a marker of bacterial translocation in the GI tract, in dogs with MMVD. We report new findings with respect to MMVD severity and presence of owner-reported GI signs. We also observed an association between disease severity and both endotoxemia and systemic inflammation, a new observation in dogs with MMVD.

A number of human and veterinary studies have described differences in circulating inflammatory cytokine concentrations between healthy individuals and individuals with CVD, as well as differences in those with early stages of CVD and CHF. Our noted association between increased circulating IL-6 concentrations and increased CVD severity is consistent with several human and canine reports [28,48,49,58]. In a previous study of dogs with MMVD, serum inflammatory cytokine concentrations, including TNF-α and IL-1β, along with IL-6 were also increased in relationship to MMVD stage severity [28]. In addition to mechanisms by which CVD can result in systemic inflammation, there have been multiple mechanisms described by which elevations in IL-6 contribute to MMVD progression, including decreased L-type calcium channel responsiveness, increased oxidative stress, decreased cardiomyocyte energy production and primary pump failure, and ventricular hypertrophy through STAT3 signaling and fibrosis [50]. These findings suggest the possible benefit to consider specific anti-inflammatory therapeutics in dogs with MMVD and elevated serum IL-6 concentrations. Although not evaluated in this study, dietary inclusion or independent supplementation with omega-3 fatty acids or similar nutrients has been evaluated in dogs with MMVD for their anti-inflammatory effects [51,52]. Specifically, some clinical benefit on heart size, as well as serum metabolomic profiles have been described [51,52]. However, neither study specifically evaluated circulating inflammatory cytokines. Our study supports the need to further investigate inflammatory pathways by which these supplements could have beneficial effects and support IL-6 as a possible parameter to monitor with supplementation, as these mechanisms have been more clearly delineated in rodent cardioprotection models [53]. The above-mentioned study also demonstrated changes consistent with cardiac remodeling, specifically increased left ventricular diastolic volume and correlation with IL-6 [28]. This is similar to humans where circulating IL-6 concentrations were reported to be higher in individuals with more severe disease and predicted hemodynamic changes in individuals with CHF, as indicated by left ventricular end-systolic volume, and contributions to systolic dysfunction have been noted in other human studies [48,49]. Whereas we utilized the LA:Ao as a measure of cardiac enlargement [54,55] and did not observe a correlation with IL-6 in our study population. However, LA:Ao is a measure of structural change, rather than functional assessment. The lack of association warrants utilization of multiple measures to assess severity of heart disease and consideration of functional echocardiographic parameters, such as ejection fraction or transmitral flow patterns. Given concerns that systemic inflammation might contribute to progression of MMVD, earlier changes in functional measurements might be more appropriate to identify early mechanisms for MMVD progression. There are also some likely differences in manifestations of cardiac disease in rodents, humans, and dogs that contribute to different phenotypic abnormalities in different populations. In rodent models, IL-6 contributes to increased collagen deposition within the cardiac muscle, contributing to ventricular hypertrophy and stiffness [56]. However, left ventricular hypertrophy is a less common finding in dogs with MMVD [57].

Importantly, the finding of increased IL-6 concentrations with increasing MMVD severity and differences reported in other circulating cytokines are not consistent across the literature. This is likely reflective of population variability, where some canine studies include a mixed disease population (i.e., dogs with MMVD and those with dilated cardiomyopathy) or evaluate single-breed versus mixed-breed populations [28,29,58,59]. In some mixed study populations, as they relate to disease or breed, no differences were noted in circulating inflammatory cytokine concentrations between disease groups [58], which would be consistent with our findings for most cytokines evaluated in the present study. Indeed, humans with DCM and CHF have increased circulating IL-2 concentrations [60], a finding which is not consistently observed in individuals with coronary artery disease [61]. Disease variability may be true for the canine population, as well. For example, a study by Zois et al [29] demonstrated a negative correlation between circulating IL-2 concentrations and increasing LA size but increased IL-8 concentrations in conjunction with increasing disease severity in Cavalier King Charles spaniels with MMVD. That study [28] suggested possible differences in mechanisms of MMVD development in CKCS compared to other breeds [62,63] as a possible contributor to their findings. This could help explain why these changes were not observed in a mixed-breed study population such as ours. Furthermore, increased circulating IL-8 concentrations were observed in ischemia-induced myocardial disease in dogs and were suggested to be secondary to myocardial inflammatory infiltrate, an uncommon finding in dogs with MMVD [27,64,65]. Assay methods likely also contribute to variability in study findings. While the above-mentioned study [29] did not note differences in circulating IL-6 in their study population, IL-6 was quantifiable in <25% dogs in that study. Furthermore, their assay had a lower level of detection cut-off for IL-2 (6.4 pg/mL) and TNF-alpha (0.4 pg/mL) than that used in our study, which could allow detection of differences between groups at lower concentrations [29].

While other studies have included dogs that were undergoing treatment for MMVD or that were receiving medications for other conditions, we elected to include only dogs that were newly diagnosed with MMVD to avoid possible confounding impacts of medications on the GI microbiome, which could subsequently affect the GI mucosal barrier function. It is possible that this limited our ability to detect changes among MMVD severity stages because of changes in cytokine expression that occur secondary to chronic CHF. Additionally, tissue-level cytokine expression may not be reflected in serum concentrations [66]. As differences observed in other studies have been attributed to differences in the myocardial inflammatory infiltrate [67–70], presence or absence of myocardial inflammation, which was not assessed in the present study, could impact results. Furthermore, cardiac disease in humans is associated with atherosclerosis to a greater extent than is realized in dogs, which could contribute to differences in the inflammatory responses or severity of systemic inflammation described in dogs versus humans with CVD [49,71]. The consideration of whether there is a concurrent primary source of inflammation may be important in dogs. For instance, in a pacing-induced tachycardia model of CHF in dogs, neither circulating concentrations of IL-6 nor TNF-α in serum were elevated [72]. We excluded dogs from our study that had clinically relevant comorbidities that could increase systemic inflammation. In the context of dogs with multiple disease processes, it is possible that the association with heart disease and other inflammatory cytokines described in humans could be observed.

Notably, TNF-α is commonly studied in rodent models and in human CVD patients and its physiologic roles in progression of CVD and impact on disease mortality have been well-described [73]. Increased TNF-α can promote a self-perpetuating cycle of inflammation by stimulating increases in other inflammatory cytokines and subsequently further increases in TNF-α [74]. TNF-α mediated inflammation also promotes cardiac cell death [75]. In naturally occurring heart failure in humans, elevated circulating TNF-α concentrations have been associated with increased CVD mortality [76]. A role for TNF-α in canine MMVD has also been demonstrated, with increased circulating concentrations in dogs with CHF versus without [59]. TNF-α was neither increased in our MMVD population compared to healthy control dogs nor were increasing concentrations associated with MMVD severity. The reason for this lack of association is unclear. Interestingly, one human study reported more severe increases in circulating TNF-α concentrations in individuals with the most advanced disease, as manifested by cardiac cachexia [73]. Most dogs in our study (22 out of 36; 61%) were assessed to have a normal MCS and a normal or increased BCS (25 out of 36; 69%). Only 3 of 36 dogs (8%) were described as having moderate muscle atrophy, and none were scored as having severe muscle atrophy. It is possible that selecting a subset of dogs with cardiac cachexia would show an association with TNF-α. This could also suggest a benefit of focused nutritional evaluation and maintaining an adequate body condition and muscle mass in dogs with MMVD. However, the correlation with TNF-α and cardiac changes has not been observed in all populations. In one human study, IL-6 was correlated with right atrial pressure and was not impacted by the presence or absence of cachexia, which might be more applicable to our study population [77].

To the authors’ knowledge, this is the first study to prospectively evaluate the presence of GI signs in dogs with MMVD in association with disease severity. Not only did we find a higher prevalence of clinical signs of GI disease in dogs with more advanced MMVD stage, but MMVD stage was also associated with higher serum LPS concentrations. Increased serum LPS concentrations in dogs with more advanced disease suggests greater occurrence of GI microbiota translocation in dogs with worse MMVD severity. It is possible that decreased GI blood flow due to reduced cardiac output leads to disruption of the GI mucosa barrier, allowing for bacterial translocation and higher detectable circulating LPS concentrations. Although the association with bacteremia and survival has not been specifically evaluated in dogs with MMVD, it is a common reason for hospitalization in humans with heart failure and increases 30-day mortality rates [78]. Bacteremia is associated with worse clinical outcomes. Therefore, it would be of interest to compare disease progression and overall survival in dogs with Stage C MMVD with increased serum endotoxin concentrations compared to those with normal concentrations. In contrast, there was no significant difference between serum LPS concentrations in dogs with MMVD Stages B1 or B2 with normal left atrial pressure. One possibility for this lack of difference is preservation of sufficient GI blood flow to maintain the GI barrier function during earlier MMVD stages. However, while it cannot be determined from this study, it is possible that increased serum LPS concentrations could contribute to progressive MMVD and gradually progressive changes to the mucosal barrier function prior to statistically significant differences being observed. A larger study population, with ability to assess MMVD Stages B1 and early B2 could help to clarify this possibility. Additionally, as described below, temporal evaluation of GI microbiome shifts during the progression of heart disease, with specific focus on those bacterial species that maintain the gut mucosal barrier would help to further describe the interrelation among these physiological changes. Nonetheless, the finding of increased circulating LPS concentrations in dogs with more advanced MMVD warrants further investigation as to whether therapy directly aimed at maintaining the mucosal barrier function could help slow progression of MMVD. Alternatively, it is possible that circulating LPS concentrations could normalize with stabilization of disease and improved GI perfusion, warranting comparison to a group of stable CHF dogs.

Furthermore, LPS concentrations were positively correlated with IL-6 concentrations in this MMVD study population. These relationships align with prior human and veterinary literature supporting the role of the gut-heart axis in patients with CVD [50]. The association with both increased circulating LPS in individuals with CVD, as well as increasing concentrations with disease severity, is consistent with findings in human studies [15–19]. Endotoxemia induces an IL-6 response secondary to toll-like receptor binding, and elevated circulating IL-6 concentrations have been documented in experimentally induced endotoxemia in humans [79]. This would support a direct link between GI translocation in dogs with severe MMVD and the observed IL-6 systemic inflammatory response in our study population. This is also consistent with a human study demonstrating higher endotoxin concentrations in individuals with uncontrolled CHF (i.e., edema) and stable CHF, with concurrently higher IL-6 concentrations in those with uncontrolled CHF [16]. As dogs receiving treatment for CHF were excluded from this study, it would be interesting to compare IL-6 concentrations in dogs with newly diagnosed CHF to those with stable, managed CHF.

To the authors’ knowledge, this is also the first study to prospectively evaluate GI clinical signs in relation to CVD severity in dogs with MMVD. Despite the small size of the study population, we identified a greater overall occurrence of owner-reported GI clinical signs in dogs with more advanced MMVD stages utilizing questionnaires based on established severity scoring for dogs with chronic enteropathies. While cause versus effect cannot be determined based on this study design, our study supports the interrelation between the cardiovascular and GI systems, as has been described in humans. Further studies to determine the impact of microbiota-targeted therapeutics (e.g., fecal microbiota transplantation, pre- or probiotics) on the GI mucosal barrier function and progression of MMVD in this study population are needed. While much of the knowledge in this area of research is advanced in humans in comparison to veterinary medicine, the relatively short time period over which canine MMVD progresses in comparison to human CVD could allow application of such microbiome studies back to human medicine.

This study has several limitations, the first being its small sample size. While our initial sample size calculation determined the need for 25 dogs in each MMVD group, it was challenging to recruit dogs with untreated Stage C MMVD, leading to a smaller total sample size. This could have limited the ability to determine differences between CVD dogs and healthy control dogs in comparison to previous studies that included a larger number of high MMVD stage dogs in their overall population, as well as a decreased ability to determine differences in cytokine concentrations between MMVD severity stages. Ideally, a larger study population would also allow evaluation among each MMVD stage, rather than two groups, permitting closer assessment of parameter progression between stages. Additionally, ideally, the control population would have been matched for age and weight with the study population, but the control population had both a higher median weight and a younger age. That said, neither age nor weight were statistically identified as confounders in our MMVD dogs, and age, gender, or weight were also not associated with differences in cytokine concentrations in a previous study of dogs with CVD [58]. Our reported prevalence of GI signs was also based on owner reports and assessment of fecal scoring. Although this may introduce some degree of subjectivity to the results, the questionnaire was based on standardized screening for chronic enteropathies in dogs, and both the questionnaire and the fecal scoring were completed prior to echocardiogram performance to avoid investigator or owner bias in FS or describing GI clinical signs based on knowledge of MMVD severity. Finally, this study did not directly assess the gut microbiome. Several canine studies have previously described the gut microbiota changes in dogs with CVD (MMVD), with mixed results. Some of those studies have demonstrated differences in alpha and beta diversity indices, as well as gut bacterial population differences between healthy dogs and MMVD dogs, with greater abnormalities in those with more severe disease [11]. In contrast, other studies have not documented phyla level differences in the gut bacterial microbiota, as assessed by 16S rRNA sequencing, in MMVD dogs [80]. Gut microbiota and metabolome assessment in our study population could help to understand the pathophysiology underlying the GI signs in these dogs (e.g., a possible association with *C. hiranonis* and potential for bile-acid diarrhea) in addition to clarifying the observed or lack of observed systemic inflammatory responses.

In conclusion, this study demonstrated an association between GI signs, GI bacterial translocation, and systemic inflammation and disease severity in dogs with MMVD. This supports the relevance of the gut-heart axis in dogs with CVD. Future studies are needed to determine the impact of bacterial translocation or therapies to directly target intestinal dysbiosis on disease-associated survival in dogs with MMVD.

## Supporting information

S1 Questionnaire Owner Questionnaire for Screening 36 Client-owned Dogs with Untreated Myxomatous Mitral Valve Disease for Gastrointestinal Clinical Signs. (DOCX)

S2 Table Serum LPS, Cytokines, and Cardiac Troponin Concentrations in 36 Dogs with Untreated Myxomatous Mitral Valve Disease (MMVD), Including 25 with Stage B1 or B2 Disease without Increased Left Atrial Pressure (Group 1) and 11 Dogs with Stage B2 Disease and Increased Left Atrial Pressure or Stage C Disease (Group 2) Enrolled in a Prospective Study Compared to Healthy Dogs. *MMVD disease severity stage group 1: Stage B1: Mitral or tricuspid valve regurgitation but no echocardiographic remodeling [left atrial (LA) or ventricular (LV) enlargement]; Stage B2 (early): Mild to moderate LA enlargement but without increased LA pressure, normal to impaired LV filling, no congestive heart failure (CHF). Group 2: Stage B2: Moderate to severe LA enlargement with increased LA pressure, normal to impaired LV filling, no CHF; Stage C: Current or past signs of CHF caused by MVD. Data are presented as median [range]. †A Bonferroni corrected *P* value of < 0.003 was considered statistically significant. IL, interleukin; LPS, lipopolysaccharide; TNF, tumor necrosis factor.
(DOCX)
