# Supplementary material for: Dogs with advanced myxomatous mitral valve disease have evidence of gastrointestinal bacterial translocation and systemic inflammation
Source: PLoS One. 2025 Nov 24;20(11):e0337580. doi: 10.1371/journal.pone.0337580 (PMC12643309; doi:10.1371/journal.pone.0337580)
Supplement: S1 Questionnaire — (DOCX) [file pone.0337580.s001.docx]

**S1 Questionnaire. Owner Questionnaire for Screening 36 Client-owned Dogs with Untreated Myxomatous Mitral Valve Disease for Gastrointestinal Clinical Signs**

**Evaluation of Dogs for Signs for Gastrointestinal Clinical Signs**

**Study group: (circle one): B1/B2 no increased atrial pressure** or **C/increased atrial pressure** or **healthy**

**Patient Name:**

**ID Number:**

**Age:**

**Breed:**

**Gender:**

**Weight (kg):**

**Body condition score (X/9):**

**Canine IBD Activity Index^1^**

1. How do you rate your dog’s current attitude/activity level compared to their normal attitude?

A. Normal

B. Slightly decreased

C. Moderately decreased

D. Severely decreased

2. How do you rate your dog’s current appetite compared to their normal appetite?

A. Normal

B. Slightly decreased (3/4 or more of normal; will still eat normal dog food)

C. Moderately decreased (1/2-3/4 of normal; needs enticement or treats/novel food)

D. Severely decreased (<1/2 of normal to absent; little to no interest in any food source)

3. How often does your dog vomit?

A. Never (no vomiting within past four weeks)

B. 1 time or less per week

C. 2-3 times per week

D. >3 times per week

4. How would you describe your dog’s stool?

A. Normal (formed stool; able to be picked up)

B. Slightly soft; presence of mucous or blood

C. Very soft but still solid; with or without mucous or blood

D. Liquid diarrhea; with or without mucous or blood

5. What is the frequency of your dog’s defecation?

A. Normal

B. 2-3 times per day

C. 4-5 times per day

D. >5 times per day

6. How would you describe your dog’s body weight trends?

A. No change in weight or weight gain

B. Mild weight-loss (<5% body weight)

C. Moderate weight-loss (5-10% body weight)

D. Severe weight-loss (>10% body weight)

Please estimate the total pounds lost: ________lb__ and period of time: __________weeks/months if applicable

**Please answer the following questions:**

1. When was the last time your dog showed any gastrointestinal signs (vomiting, diarrhea, weight-loss, decreased appetite)? Please describe: _________________________________________________________

________________________________________________________________________________________

2. Does your dog receive Heartworm/Flea prevention? ___________ Brand? __________________________

3. Is your dog up-to-date on vaccinations? ______________________________________________________

4. What medications is your dog taking? ­_______________________________________________________

5. What food does your dog eat? _____________________________________________________________

6. What treats does your dog receive? _________________________________________________________

7. What supplements does your dog receive (e.g. glucosamine, probiotics)? ___________________________

8. What treatments has your dog received for any medical problems?

_____________________________________________________________________________________

And when were they last given? ___________________________________________________________

9. What other medical conditions does your dog have? ____________________________________________

________________________________________________________________________________________

1- Jergens AE, Schreiner CA, Frank DE, et al. A scoring index for disease activity in canine inflammatory bowel disease. *JVIM*. 2003; 17: 291-297.
