# Supplementary material for: Dogs with advanced myxomatous mitral valve disease have evidence of gastrointestinal bacterial translocation and systemic inflammation
Source: PLoS One. 2025 Nov 24;20(11):e0337580. doi: 10.1371/journal.pone.0337580 (PMC12643309; doi:10.1371/journal.pone.0337580)
Supplement: S2 Table — *MMVD disease severity stage group 1: Stage B1: Mitral or tricuspid valve regurgitation but no echocardiographic remodeling [left atrial (LA) or ventricular (LV) enlargement]; Stage B2 (early): Mild to moderate LA enlargement but without increased LA pressure, normal to impaired LV filling, no congestive heart failure (CHF). Group 2: Stage B2: Moderate to severe LA enlargement with increased LA pressure, normal to impaired LV filling, no CHF; Stage C: Current or past signs of CHF caused by MVD. Data are presented as median [range]. †A Bonferroni corrected P value of < 0.003 was considered statistically significant. IL, interleukin; LPS, lipopolysaccharide; TNF, tumor necrosis factor. (DOCX) [file pone.0337580.s002.docx]

**S2 Table**. **Serum LPS, Cytokines, and Cardiac Troponin Concentrations in 36 Dogs with Untreated Myxomatous Mitral Valve Disease (MMVD), Including 25 with Stage B1 or B2 Disease without Increased Left Atrial Pressure (Group 1) and 11 Dogs with Stage B2 Disease and Increased Left Atrial Pressure or Stage C Disease (Group 2) Enrolled in a Prospective Study Compared to Healthy Dogs.**

| Biomarkers (units) | All MVD | MVD Group 1 | MVD Group 2 | Healthy | *P* value† | | |
| --- | --- | --- | --- | --- | --- | --- | --- |
|  |  |  |  |  | **MVD vs Healthy** | **Group 1 vs Healthy** | **Group 2 vs Healthy** |
| IL-2 (pg/mL) | 69  [20-351] | 69  [20-215] | 50  [20-351] | 64  [20-140] | 0.689 | 0.609 | 0.973 |
| IL-6 (pg/mL) | 61  [12-251] | 55  [12-176] | 106  [14-251] | 53  [13-102] | 0.423 | 0.653 | 0.223 |
| IL-8 (pg/mL) | 3231  [144-12242] | 3484  [198-11504] | 1603  [144-12242] | 2523  [784-5315] | 0.803 | 0.397 | 0.349 |
| LPS (ng/mL) | 6.4  [1.3-12.4] | 5.8  [1.3-12.4] | 9.3  [4.3-11.3] | 7.4  [3.7-10.8] | 0.768 | 0.756 | 0.092 |
| TNF-α (pg/mL) | 6.7  [4.8-41.2] | 7.9  [4.8-25.3] | 4.8  [4.8-41.2] | 6.7  [4.8-14.7] | 0.544 | 0.369 | 0.892 |
| Troponin (pg/mL) | 96  [20-1610] | 52  [20-198] | 371  [111-1610] | 25  [20-76] | **<0.001** | 0.012 | **<0.0001** |

*MMVD disease severity stage group 1: Stage B1: Mitral or tricuspid valve regurgitation but no echocardiographic remodeling [left atrial (LA) or ventricular (LV) enlargement]; Stage B2 (early): Mild to moderate LA enlargement but without increased LA pressure, normal to impaired LV filling, no congestive heart failure (CHF). Group 2: Stage B2: Moderate to severe LA enlargement with increased LA pressure, normal to impaired LV filling, no CHF; Stage C: Current or past signs of CHF caused by MVD.

Data are presented as median [range]. †A Bonferroni corrected *P* value of < 0.003 was considered statistically significant

IL, interleukin; LPS, lipopolysaccharide; TNF, tumor necrosis factor
